# Structural Implications of H233L and H398P Mutations in Phospholipase Cζ: A Full-Atom Molecular Dynamics Study on Infertility-Associated Dysfunctions

**DOI:** 10.3390/ijms26104706

**Published:** 2025-05-14

**Authors:** Fernando Hinostroza, Sofía Albornoz-Muñoz, Sebastián Vergara, Gabriela Urra, Ingrid Araya-Durán, Rafael A. Fissore, Fernando Danilo González-Nilo, Daniel Bustos, Ingrid Carvacho

**Affiliations:** 1 Centro de Investigación de Estudios Avanzados del Maule (CIEAM), Vicerrectoría de Investigación y Postgrado, Universidad Católica del Maule, Talca 3460000, Chile; 2 Centro de Investigación en Neuropsicología y Neurociencias Cognitivas (CINPSI Neurocog), Facultad de Ciencias de la Salud, Universidad Católica del Maule, Talca 3460000, Chile; 3 Departamento de Medicina Traslacional, Facultad de Medicina, Universidad Católica del Maule, Talca 3460000, Chile; sebadiantian@gmail.com; 4 Centro para la Investigación Traslacional en Neurofarmacología, Universidad de Valparaíso, Valparaíso 2340000, Chile; 5 Escuela de Ingeniería en Biotecnología, Facultad de Ciencias Agrarias y Forestales, Universidad Católica del Maule, Talca 3460000, Chile; sofiaalbornozhb@gmail.com; 6 Laboratorio de Bioinformática y Química Computacional, Departamento de Medicina Traslacional, Facultad de Medicina, Universidad Católica del Maule, Talca 3460000, Chile; gabriela.urra@alu.ucm.cl (G.U.); dbustos@ucm.cl (D.B.); 7 Center for Bioinformatics and Integrative Biology (CBIB), Universidad Andrés Bello, Santiago 8370146, Chile; ingrid.araya.duran@gmail.com (I.A.-D.); danilo.gonzaleznilo@gmail.com (F.D.G.-N.); 8 Department of Veterinary and Animal Sciences, University of Massachusetts, Amherst, MA 01003, USA; rfissore@vasci.umass.edu

**Keywords:** Phospholipase Cζ, phosphatidylinositol 4,5-bisphosphate, infertility, H233L mutation, H398P mutation, R553P mutation

## Abstract

Phospholipase Cζ (PLCζ), a sperm-specific enzyme, plays a critical role in mammalian fertilization. Mutations in PLCζ have been linked to male infertility, as they impair its ability to trigger calcium (Ca^2+^) oscillations necessary for egg activation and embryo development. During fertilization, PLCζ is introduced into the egg, where it hydrolyzes phosphatidylinositol 4,5-bisphosphate (PIP_2_) into inositol 1,4,5-trisphosphate and diacylglycerol, leading to Ca^2+^ release from the endoplasmic reticulum. Human infertility-associated mutations include H233L, H398P, and R553P, which disrupt PLCζ function. To elucidate the molecular consequences of the mutations, we employed full-atom molecular dynamics simulations to analyze structural perturbations and their impact on PIP_2_ and Ca^2+^ binding. Our results reveal that H233L and H398P mutations significantly reduce interactions with PIP_2_, disrupting hydrogen bonding and salt bridge formation, leading to misalignment of the substrate. Additionally, these mutations destabilize Ca^2+^ binding by altering its positioning within the active site. In contrast, the R553P mutation primarily affects intramolecular stability and enzyme dynamics without impairing substrate or ion binding. Free energy calculations indicate an increased affinity for PIP_2_ in H233L and H398P mutants, leading to an aberrant substrate positioning and compromised hydrolysis. These structural insights help explain the egg activation failure and infertility of patients carrying these mutations.

## 1. Introduction

Infertility affects millions of men and women of reproductive age. Worldwide, infertility affects ~15% of couples, and 7% of males suffer from infertility. Male infertility accounts for ~30% of the total infertility cases, and genetic defects cause male infertility, including mutations in PLCΖ1 [1,2,3], which encodes Phospholipase Cζ (PLCζ).

**Figure 1:**
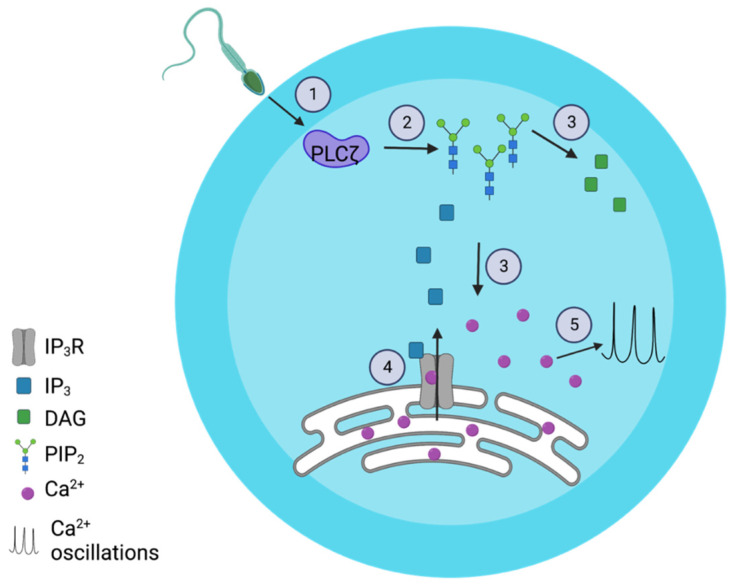
PLCζ induces Ca^2+^ oscillations in the egg. 1. Sperm fuses with the egg and releases its genetic material and the PLCζ into the egg. 2. PLCζ binds PIP_2_. 3. PLCζ hydrolyzes PIP_2_ to produce IP_3_ and DAG. 4. IP_3_ binds IP_3_ receptors in the endoplasmic reticulum and induces Ca^2+^ release. 5. The release of intracellular Ca^2+^ triggers Ca^2+^ oscillations and activates the egg.

PLCζ (PLCZ1) is a sperm-specific enzyme that triggers calcium (Ca^2+^) oscillations and egg activation [4,5]. Following release into the egg upon fusion, PLCζ hydrolyzes phosphatidylinositol 4,5-bisphosphate (PIP_2_), associated with cellular membranes and vesicles, producing inositol 1,4,5-triphosphate (IP_3_) [4,6,7]. IP_3_ binds IP_3_ receptors, the most common in eggs, in type 1 (IP3R1), inducing Ca^2+^ release from the endoplasmic reticulum (ER) and periodic intracellular Ca^2+^ concentration (Ca^2+^) elevations, known as Ca^2+^ oscillations [8,9] (Figure 1). Sperm derived from mice lacking PLCζ1 (*Plcz1*^−/−^) fail to induce Ca^2+^ oscillations [10] or show an impaired pattern of them with a decreased number of oscillations compared with WT sperm [11]. *Plcz1*^−/−^ sperm fertilization results in polyspermy when fertilizing eggs in vivo. Consequently, these findings confirm the essential role of the Ca^2+^ oscillations in modulating the block of polyspermy [10]. The fertility of *Plcz1*^−/−^ males is also greatly affected, and the animals exhibit a subfertile phenotype. However, *Plcz1*^−/−^ animals are not sterile, suggesting a PLCζ1-independent fertility route [10,11]. Moreover, PLCζ mutations identified in a cohort of couples undergoing fertility treatment were replicated in mouse models to assess their impact on early embryonic cleavage and pregnancy outcomes. The authors reported lower rates of cell division, impaired embryo quality, lower pregnancy success, and smaller litter sizes following IVF using sperm from *Plcζ* mutant males with WT eggs, compared to IVF with WT *Plcζ* sperm [12]. Therefore, the PLCζ function is required for the egg-to-embryo transition. In fact, suppression or failure to initiate Ca^2+^ oscillations causes egg activation failure and impairs early embryo development [13,14].

**Figure 2:**
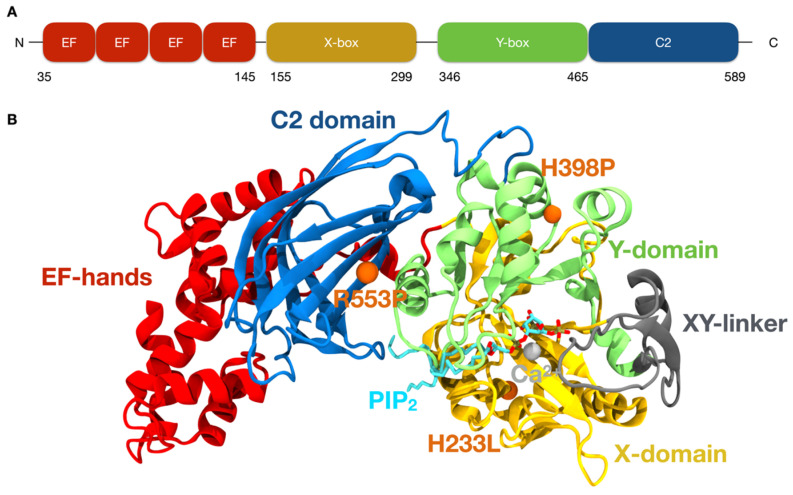
PLCζ structure. (**A**) The N-terminus of the PLCζ has an EF-hand domain, and X- and Y-domains linked by an XY linker, and a C2 domain in its C-terminus. (**B**) AlphaFold model of the human PLCζ binding PIP_2_ and Ca^2+^.

Structurally, PLCζ has two EF-hand domain pairs in its N-termini, a catalytic domain composed of X and Y domains connected by a loop called X-Y linker, and a C2 domain in its C-termini region [4,15] (Figure 2A,B). Unlike other cellular PLCs, PLCζ lacks an N-terminus PH domain. The integrity of the four EF-hand domains and the C-terminal C2 domain is essential for PLCζ to reach its maximal activity [16], while the catalytic domain is involved in PIP_2_ hydrolysis facilitated by Ca^2+^ binding [15,17]. The X-Y linker is proposed to interact with the plasma membrane (PM) and PIP_2_ [17,18].

Several mutations in PLCζ have been related to male infertility [2,3,19,20]. They are generally located in the catalytic domain (X or Y), but some have been identified in the C2 domain. In fact, the mutation I489F located in the C2 domain is linked to infertility in humans. In vitro studies demonstrated that microinjection of the mutant protein at physiological concentrations failed to induce Ca^2+^ oscillations or support embryo development. Interestingly, the phenotype could be rescued by increasing the concentration of the recombinant I489F mutant protein. Biochemical analyses revealed that the mutation does not impair enzymatic activity or the Ca^2+^ sensitivity of the PLCζ. However, I489F significantly disrupts the binding of PLCζ to PI(3)P and PI(5)P_,_ underlying the infertility phenotype reported in the patient [21]. Only one of the mutations is in the EF domain [5]. The mutations H233L and H398P, which comprised exchanges of histidine for leucine in position 233 [2] (Figure 2B) and for proline in position 398 [13]. Sperm from patients carrying the mutation H233L were injected into human eggs, and oocyte activation was evaluated, showing a lower percentage of activation than WT oocytes [20]. PLCζ1 mutation H398L patients are infertile, and in the mouse oocyte activation test (MOAT), injection of the mutated cRNA showed an abnormal pattern of Ca^2+^ oscillations [13]. The mutations were initially found in the same patient as compound heterozygous mutations with maternal and paternal inheritance, respectively. The H233L mutation is located in the X-domain, part of the catalytic site (Figure 2) [2,20], and H398P in the Y-box domain (Figure 2) [13]. This patient also seemed to have reduced expression of PLCζ [1]. A more recent homozygous mutation was described in the PLCζ C2 domain, R553P, where arginine was exchanged for proline (Figure 2). Injection of R553P Plcζ cRNA into mouse oocytes failed to activate eggs and trigger embryo development [22]. Although PLCZ1 mutations are individually rare, their clinical relevance is substantial. Notably, 33.6% of men who experience fertilization failure after intracytoplasmic sperm injection (ICSI) have been found to carry *PLCZ1* variants [23], highlighting the importance of genetic screening in assisted reproduction settings.

Despite the obvious impact on fertility, it remains elusive how these mutations affect the structure of PLCζ and its association with PIP_2_ at a molecular level. Here, we performed long-timescale full-atom molecular dynamics (MD) simulations to understand at an atomic level how these mutations located in different domains impact the structure of PLCζ and the binding of its natural ligand, PIP_2_. Our data showed that the H233L and H398P displayed fewer hydrogen bonds and salt bridges between PLCζ and PIP_2_, reduced total contacts and binding energy, suggesting a destabilization of the PLCζ-PIP_2_ complex. Moreover, the simulations indicated that these mutations modified PLCζ Ca^2+^ ion binding. On the other hand, the R553P mutation affects intramolecular dynamics and possibly membrane-binding. In conclusion, our results showed that H233L and H398P mutations modified the structure of PLCζ, triggering a shift in the position of PIP_2_ and Ca^2+^. Therefore, preventing PIP_2_ hydrolysis. Our data provide relevant insights into the structure of PLCζ and the mechanisms by which its function is precisely regulated, highlighting its fundamental role in human fertility.

## 2. Results

### 2.1. H233L and H398P Mutations Altered PIP_2_ Binding

**Figure 3:**
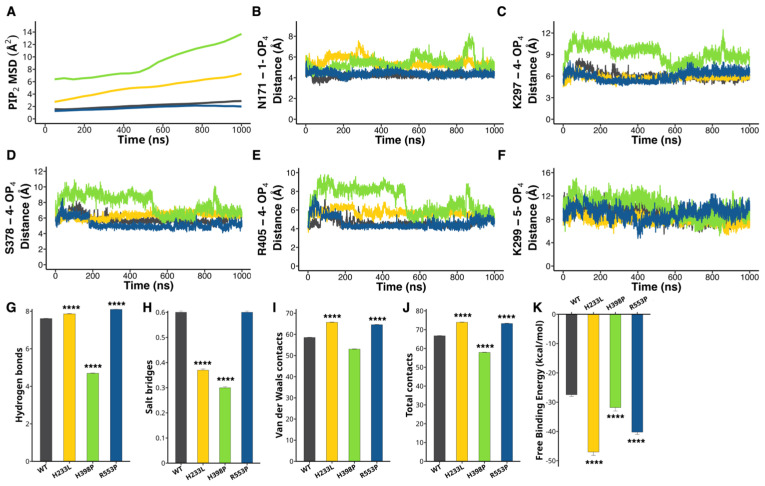
H233L and H398P mutations impair PIP_2_ binding. MD of the PLCζ mutants H233L (yellow), H398P (green), and R553P (blue) in comparison with WT (black) are evaluated using structural and functional parameters. (**A**) Mean Square Deviation (MSD) of PIP_2_ during the simulation. (**B**) Average distance of N171 with 1-OP4. Distance of (**C**) K297, (**D**) S378, and (**E**) R405 with 4-OP4. (**F**) Average distance of K299 with 5-OP4. Amount of (**G**) hydrogen bonds, (**H**) salt bridges, (**I**) van der Waals, and (**J**) total contacts of PIP_2_ with PLCζ. (**K**) Free binding energy of PIP_2_ with PLCζ. **** *p* < 0.0001.

The injection of H233L, H398P, and R553P PLCζ mRNA mutants or the presence of these mutations in human patients fails to activate eggs and causes infertility [2,20,22]. To assess whether this failure alters PIP_2_ binding, we measured the Mean Square Deviation (MSD) of PLCζ for PIP_2_, which measures the average squared distance that the molecules travel over time within the binding site. The average PIP_2_ MSD values for the H233L and H398P mutants were higher than those for R553P and WT (Figure 3A), indicating greater molecular spatial dispersion and instability during the simulated interaction (H233L: 5.11 ± 0.28 Å^2^, H398P: 9.11 ± 0.54 Å^2^, R553P: 1.80 ± 0.06 Å^2^, WT: 2.18 ± 0.09 Å^2^). Notably, despite the inherent flexibility of the PIP_2_ acyl chains, the overall MSD values in WT and R553P remained low, indicating stable binding within the active site. This suggests that the lateral chain mobility does not significantly affect the MSD measurement, supporting the validity of our approach without requiring a separate analysis of the myo-inositol bisphosphate moiety.

To further comprehend the deviation observed for the mutants H233L and H398P, we calculated the distance of amino acids involved in PIP_2_ stability. We observed that the N171, which interacts with the 1-OP4, exhibits an average distance of 4.23 ± 0.003 Å for the WT and 4.38 ± 0.003 Å for the R553P mutant. In contrast, the H233L and the H398P mutants showed a larger distance, being 5.47 ± 0.006 Å and 5.39 ± 0.008 Å, respectively (Figure 3B). The K297, S378, and R405 stabilize the 4-OP4. The average distance for the K297 in the WT enzyme was 6.06 ± 0.08 Å, and 5.77 ± 0.004 Å, 9.11 ± 0.01 Å, and 5.99 ± 0.009 Å for the H233L, H398P, and R553P mutants, respectively (Figure 3C). For the S378, the average distance was 5.88 ± 0.007 Å, 6.3 ± 0.004 Å, 7.8 ± 0.01 Å, and 5.21 ± 0.008 Å for the WT, H233L, H398P, and R553P proteins, respectively (Figure 3D). The average distance for the R405 in the WT and the R553P enzymes was 4.63 ± 0.006 Å and 4.61 ± 0.008 Å. In contrast, the H233L and the H398P mutants exhibited 5.67 ± 0.005 Å and 7.08 ± 0.01 Å (Figure 3E). The K299 contacts the 5-OP4 and in the WT enzyme has an average distance of 9.03 ± 0.01 Å. In contrast, the H233L, H398P, and the R553P mutants 7.98 ± 0.01 Å, 10.16 ± 0.02 Å, and 9.06 ± 0.01 Å, respectively (Figure 3F). These results indicate that the mutation H233L and the H398P in PLCζ impair PIP_2_ binding, favoring mispositioning of PIP_2_, which would prevent its effective hydrolysis and cause failure to initiate oscillations.

We also analyzed the types of contacts between PLCζ and PIP_2_, including hydrogen bonds, salt bridges, and van der Waals contacts. In the WT complex, PIP_2_ formed an average of 7.6 ± 0.01 hydrogen bonds with WT PLCζ, whereas the H233L complex formed 7.8 ± 0.02 bonds (**** *p* < 0.0001), the H398P complex formed 4.6 ± 0.01 bonds (**** *p* < 0.0001), and the R553P complex formed 8.1 ± 0.01 bonds (**** *p* < 0.0001), all statistically different from the WT (Figure 3G). Salt bridges were less frequent in H233L and H398P (0.37 ± 0.005, 0.3 ± 0.004, respectively, **** *p* < 0.0001), but R553P and WT were comparable (0.6 ± 0.006) (Figure 3H). On the contrary, van der Waals’ contacts were more frequent for H233L (65.7 ± 0.1, **** *p* < 0.0001) and R553P mutant (64.5 ± 0.1, **** *p* < 0.0001) compared to H398P (53 ± 0.1) and WT PLCζ (58.5 ± 0.08) (Figure 3I). However, because of the variation in type and number of interactions with PIP_2_, we calculated the total bonds formed between PLCζ and PIP_2_ for the different PLCζ under consideration. We found that the WT protein had a total of 66.7 ± 0.09 contacts, whereas H233L, H398, and R553P mutants displayed 73.9 ± 0.1, 57.9 ± 0.1, and 73.29 ± 0.1 contacts, respectively (**** *p* < 0.0001; Figure 3J).

Next, we compared the free binding energy of PIP_2_ to PLCζ. The WT complex exhibited a mean binding energy of −27.43 ± 0.5 kcal/mol. In contrast, the average binding energy for the H233L, H398P, and R553P was −47 ± 1.1 kcal/mol, −31.8 ± 1.2 kcal/mol, and −40.22 ± 0.7 kcal/mol, respectively (**** *p* < 0.0001; Figure 3K). Lastly, we evaluated the PIP_2_ diffusion coefficient and found higher values for the H233L and the H398P mutants vs. the WT and the R553P mutant (Figure S1A).

### 2.2. H233L and H398P Mutations Impair Ca^2+^ Binding

**Figure 4:**
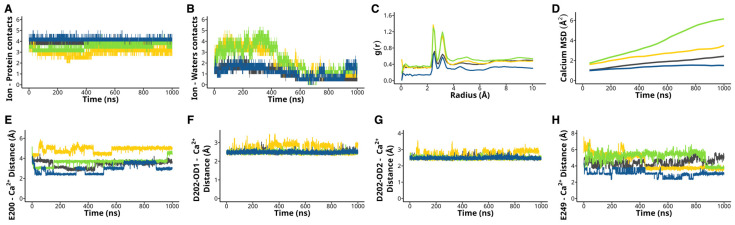
H233L and H398P mutations modify Ca^2+^ binding. The Ca^2+^ binding dynamics for the PLCζ mutants H233L (yellow), H398P (green), and R553P (blue) are analyzed by the evaluation of Ca^2+^ contacts and hydration. (**A**) Amount of Ca^2+^ contacts with PLCζ. (**B**) Amount of Ca^2+^ contacts with water molecules. (**C**) Radial distribution function (g(r)) of water molecules around the Ca^2+^ ion. (**D**) The Ca^2+^ Mean square deviation (MSD) throughout the simulation. Average distance of (**E**) E200, (**F**) D202-OD1, (**G**) D202-OD2, and (**H**) E249 with Ca^2+^.

Ca^2+^ ions play a crucial role in facilitating PIP_2_ binding by PLCζ [15]. Therefore, we analyzed whether H233L, H398P, and R553P mutations modify Ca^2+^ binding to PLCζ. Our analysis revealed that the WT PLCζ formed, on average, 3.8 ± 0.005 contacts with Ca^2+^, whereas the H233L and H398P mutations had fewer amino acid contacts, 3.0 ± 0.008 and 3.4 ± 0.005, respectively. The R553P mutant had an average of 4.1 ± 0.002 contacts with Ca^2+^ throughout the simulation (Figure 4A).

We also measured the number of contacts the Ca^2+^ ion made with water molecules. Throughout the simulation, we found that all systems showed a reduction in the number of water contacts, with the WT PLCζ having an average of 1.0 ± 0.006 and the R553P having 1.1 ± 0.007 contacts, whereas the H233L had 2.1 ± 0.01 and H398P had 2.2 ± 0.01 contacts (Figure 4B). Notably, during the first half of the simulation, the H233L and H398P systems established nearly twice the number of solvation contacts with Ca^2+^ compared to the other systems. To complement this analysis, we quantified the density of water molecules around Ca^2+^ by measuring the radial distribution function (RDF, g(r)). The peaks indicate preferred distances where water molecules are more likely to be found, thus reflecting the hydration shell and interaction dynamics between the ion and the surrounding water. We found that the systems had two peaks around 2.55 and 3.15 Å RDF, larger for H233L and H398P than for R553P and the WT protein (Figure 4C).

Further, we found that the MSD of Ca^2+^ throughout the simulation in the WT PLCζ had a displacement of 1.7 ± 0.1 Å^2^. In contrast, the H233L, H398P, and R553P mutations exhibited MSDs of 2.5 ± 0.07, 3.9 ± 0.2, and 1.3 ± 0.03, respectively (Figure 4D). Consistent with these values, the Ca^2+^ ion’s diffusion coefficient for the mutations H233L and H398P showed a higher value than the one for the R553P mutation and the WT complex (Figure S1B). Specifically, the mean diffusion coefficients for H233L (0.000319 ± 0.0000940 Å^2^/ns) and H398P (0.000443 ± 0.0000485 Å^2^/ns) were nearly double those of the R553P (0.000173 ± 0.0000688 Å^2^/ns) and WT (0.000222 ± 0.0000541 Å^2^/ns) complexes, indicating greater ion mobility in these two mutants.

Additionally, we calculated the distance of E200, D202, and E249, which stabilizes the Ca^2+^. The average distance of the E200 in the WT enzyme was 3.42 ± 0.004 Å, whereas for the H233L, H398P, and the R553P mutants, it was 4.87 ± 0.004 Å, 3.63 ± 0.004 Å, and 2.92 ± 0.005 Å, respectively (Figure 4E). We also measured the average distance of both oxygens (OD1 and OD2) bonded to the gamma carbon of D202, which interacts with Ca^2+^. For the D202-OD1, the WT average distance was 2.47 ± 0.001 Å, for the H233L enzyme was 2.62 ± 0.003 Å, for the H398P mutant was 2.46 ± 0.0008 Å, and for the R553P protein was 2.47 ± 0.0008 Å (Figure 4F). For the D202-OD2, the average distance of the WT PLCζ was 2.48 ± 0.001 Å, whereas for the H233L, H398P, and the R553P mutants, it was 2.62 ± 0.003 Å, 2.47 ± 0.0008 Å, and 2.48 ± 0.0008 Å, respectively (Figure 4G). For the E249, the distance was 4.43 ± 0.006 Å for the WT, 4.48 ± 0.01 Å for the H233L mutant, 5.12 ± 0.008 Å for the H398P enzyme, and 3.05 ± 0.005 Å for the R553P protein (Figure 4H). These results suggest that the H233L and H398P mutations modify PLCζ Ca^2+^ ion binding, impairing PIP_2_ binding and hydrolysis.

### 2.3. The H233L, H398P, and R553P Mutations Changes the PLCζ Intramolecular Interactions

**Figure 5:**
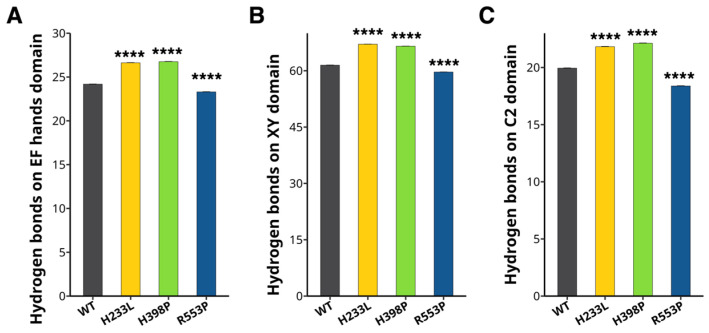
H233L and H398P changes intramolecular hydrogen bonds of PLCζ. Hydrogen bonds are analyzed in the PLCζ mutants H233L (yellow), H398P (green), R553P (blue), and WT (black). Amount of intramolecular hydrogen bonds in the (**A**) EF hands, (**B**) XY, and (**C**) C2 domains. **** *p* < 0.0001.

Hydrogen bonds are crucial non-bonded interactions that stabilize protein structures [24]. Thus, we calculated the number of hydrogen bonds to determine whether the PLCζ mutations modify the number of hydrogen bonds present in each domain (EF-hands, XY, and C2 domains). The WT EF hands domain had an average of 24.1 ± 0.03 hydrogen bonds, whereas the H233L mutation had 26.6 ± 0.03, the H398P had 26.7 ± 0.03, and the R553P mutant had 23.3 ± 0.03 (Figure 5A). The WT XY domain exhibits an average of 61.4 ± 0.06 hydrogen bonds. In contrast, the H233L, H398P, and R553P mutants had 67.0 ± 0.05, 66.5 ± 0.05, and 59.6 ± 0.05 hydrogen bonds, respectively (Figure 5B). The WT C2 domain showed 19.9 ± 0.03 hydrogen bonds, whereas the H233L, H398P, and the R553P mutants exhibited 21.8 ± 0.03, 22.1 ± 0.03, and 18.3 ± 0.03 hydrogen bonds, respectively (Figure 5C).

**Figure 6:**
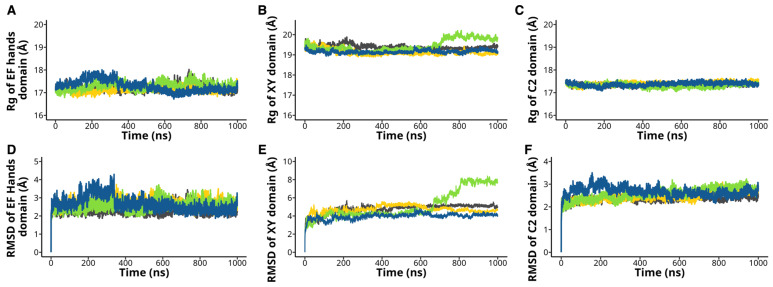
H233L, H398P, and R553P mutations did not affect the overall PLCζ structure. The radius of gyration (Rg) of (**A**) EF hands, (**B**) XY, and (**C**) C2 domains was analyzed for the PLCζ mutants H233L (yellow), H398P (green), and R553P (blue) and WT (black). Root mean square deviation (RMSD) of (**D**) EF hands, (**E**) XY, and (**F**) C2 domains was also determined for the mutants and WT proteins.

To explore whether the H233L, H398P, and R553P mutations influence the overall protein structure, we measured the radius of gyration (Rg) of each PLCζ. The Rg measures the compactness and overall shape of the enzyme. We found no significant difference between the WT and the mutant PLCζ in the Rg of the EF-hands domain and C2 domain (Figure 6A,C). On the contrary, the XY domain of the H398P exhibited a bigger Rg during the last 300 ns of simulation (Figure 6B). The Rg of PIP_2_ of the WT complex was similar to the H233L and R553P mutants but slightly bigger for the H398P mutant during the last 100 ns of simulation (Figure S2A). Then, we calculated each domain’s Root Mean Square Deviations (RMSD) for the four different conditions. RMSD quantifies the enzyme’s overall conformational changes and structural stability during the simulation. There was no significant difference between the RMSD of WT compared to the H233L and R553P mutants (Figure 6D,F). In contrast, the XY domain of the H398P mutant showed a bigger RMSD with a mean value of 5.12 ± 1.52 Å, indicating greater structural deviation and instability. This is particularly evident when compared to the WT protein (4.77 ± 0.517 Å), which maintained more consistent structural integrity (Figure 6E). Our data showed that the H233L, H398P, and R553P mutations do not change the overall structure of PLCζ but change the intramolecular interactions mediated by hydrogen bonds.

## 3. Discussion

Infertility is a complex condition affecting millions worldwide, with a significant proportion attributed to male factors [25]. Among the genes implicated in male infertility, PLCζ has emerged as a critical player due to its pivotal role in initiating egg activation through Ca^2+^ oscillations during fertilization. However, the molecular mechanism underlying the effects of specific mutations on PLCζ function remains poorly understood. Point mutations in the PLCζ gene can induce structural alterations in the protein, impacting male fertility. Despite their significance, the structural and functional consequences of such mutations have been poorly characterized. In this work, we employed MD simulations to examine the impact of three PLCζ infertility-causing mutations, H233L, H398P, and R553P, on its structure and interactions with its natural ligands PIP_2_ and Ca^2+^. By employing molecular dynamics simulations in an aqueous environment, we aimed to isolate and analyze the intrinsic structural effects of these mutations to further explain infertility in the patients carrying these changes. Our results shed light on the structural alterations induced by these mutations and provide insights into their impact on PLCζ function.

Once the sperm fuses with the egg oolemma, and PLCζ enters the ooplasm, the enzyme hydrolyzes its substrate PIP_2_ located in cellular membranes [4,6,7]. The active site structure and main residues are conserved in the PLC family and among mammals, including PLCζ [26]. The structure of PLC∂1’s active site, solved by Essen et al., [15] displays 64% identity with the active site of hPLCζ1, indicating a similar PIP_2_ binding mode and hydrolysis [4]. PLCζ binds and stabilizes PIP_2_ before hydrolysis by interacting with the 4- and 5-phosphates of the inositol group through the amino acids K299, K327, S378, R405, and Y407 and by interacting with the 2-OH and 3-OH groups with the residues D202, E249, and R405. Based on the evidence of increased MSD of PIP_2_ and altered protein-ligand contacts, including fewer hydrogen bonds and salt bridges between the enzyme and PIP_2_ for the H233L and H398P PLCζ mutants compared to the WT PLCζ, our model predicts impaired PIP_2_ binding and a destabilization of the PLCζ-PIP_2_ complex. These structural changes likely interfere with the proper positioning of PIP_2_ for hydrolysis, thereby impairing the generation of IP_3_ production, Ca^2+^ oscillations, and egg activation.

PLCζ is ~100-fold more sensitive to Ca^2+^ than PLC∂1 and is the most Ca^2+^-sensitive PLC enzyme [27]. Ca^2+^ plays an important role in the PLCζ-PIP_2_ binding since it lowers this interaction’s pKa [28,29]. Ca^+2^ is stabilized by the amino acids E200, D202, and E249, and PIP_2_ hydrolysis is produced by a nucleophilic attack of H215 on the 1-phosphate, producing DAG and IP_3_. In the H233L and H398P mutants, we observed a shift in the positions of PIP_2_ and Ca^2+^ in the PLCζ’s catalytic site, which is supported by the changes in MSD, the number of amino acids contacting PIP_2_ and Ca^2+^, and the RDF of the water molecules around Ca^2+^. Surprisingly, the free binding energy of the three mutants is lower than that of the WT, suggesting that the mutant PLCζs bind PIP_2_ with more affinity. However, the higher affinity appears to favor incorrect binding, diminishing PIP_2_ hydrolysis and, ultimately, preventing Ca^2+^ oscillations. This agrees with the fact that the PLCζ H233L, H398P, and R553P mutations reduce or suppress egg activation in humans [2,20,22]. Remarkably, H233L and H398P are not located within the enzyme’s active site but adjacent to it. However, these mutations appear to induce allosteric perturbations that disrupt PIP_2_ and Ca^2+^ binding. Specifically, 1-OP4 is displaced from N171, while 4-OP4 is repositioned away from K297, S378, and R405. Additionally, Ca^2+^ is displaced from its stabilizing residues E200 and D202, further compromising enzymatic function. This mutation-induced allosteric changes that are extended through the protein, impacting critical binding sites, have also been observed in other enzymes and proteins [30,31].

Interestingly, the R553P mutation did not significantly affect PIP_2_ or Ca^2+^ binding. These results agree that C2 domain deletion does not affect PLCζ enzymatic activity or Ca^2+^ sensitivity [32,33]. This mutation changes the residue cross-correlation patterns, suggesting a disruption of intramolecular interactions within PLCζ. However, the overall protein structure remained largely unaffected by the mutation, as indicated by similar Rg and RMSD values compared to the WT. The C2 domain is proposed to mediate the interaction with phospholipids present in the membrane, facilitating PLCζ’s adequate positioning and possible access to substrates [32,33]. Noteworthy, a homozygous I498F mutation in the C2 domain of hPLCζ impaired its distribution in eggs after mRNA injection and in the sperm before fertilization [19]. Thus, it is possible that the observed intramolecular changes could affect the C2 domain amino acids disposition and, subsequently, its binding to membranes. However, the exact mechanism by which PLCζ interacts with membranes remains unclear, highlighting the need for further research to elucidate this process.

Mice deficient in Plcζ1 (*Plcζ*^−/−^) show impaired Ca^2+^ oscillations, polyspermy, and subfertility, suggesting a compensatory or “backup” mechanism to support successful fertilization in the absence of Plcζ1 [10,11]. The egg expresses various isoforms of PLCs, including PLCβs, PLCγs, and PLCδs. While these isoforms have been reported to modulate Ca^2+^ oscillations following fertilization, none can substitute for the essential role of Plcζ in initiating fertilization. For example, overexpression of PLCβ1 in mouse eggs modifies the duration of the sperm-induced Ca^2+^ oscillations’ first transient and decreases the frequency of the oscillations [34].

Male infertility rates are rising worldwide [35], impacting the mental health of millions of men by reducing self-esteem and inducing a sense of loss [36]. Mutations in *PLCZ1* are increasingly recognized as a significant genetic cause of male infertility, particularly in individuals presenting with fertilization failure following assisted reproductive techniques such as intracytoplasmic sperm injection (ICSI). Recent studies have reported a prevalence of *PLCZ1* variants as high as 33.6% in men experiencing ICSI failure, underscoring the clinical relevance of this gene in reproductive diagnostics [23]. In clinical practice, *PLCZ1* mutations are typically identified using high-throughput genetic screening methods [13,37,38]. Whole-exome sequencing (WES) is a widely used tool, offering a comprehensive analysis of all coding regions and enabling the identification of known and novel variants. WES is particularly valuable in cases of idiopathic infertility, where there is no prior suspicion of specific genetic defects. Genetic analysis not only supports diagnosis but also guides clinical decision-making in the context of assisted reproductive technologies (ART), including the potential use of oocyte activation protocols or microinjection of wild-type *PLCZ1* mRNA. The microinjection of PLCζ into oocytes can induce Ca^2+^ oscillations needed to start egg activation and early embryo development, which has been demonstrated even in eggs previously injected with PLCζ carrying mutations [39].

## 4. Materials and Methods

### 4.1. Full-Atom Molecular Dynamics Simulations and Docking

We used the freely available human PLCζ model predicted by AlphaFold (Alpha-Fold Entry: Q86YW0) [40]. For this, we used as a reference the crystallographic structure of PLC∂ bonded to myo-inositol (PDB ID: 1DJZ) [15] since the active site amino acids are conserved between both enzymes and share a 51% identity. We use the structure of PLCζ in solution rather than at the lipid (membrane)-water interface to decrease the computational cost. Additionally, it has not been reported that PLCζ interacts with membranes. The Ca^2+^ ion was positioned between the residues N171, E200, D202, E249, and the PIP_2_ ligand. Then, we used the CHARMM-GUI website to mutate PLCζ and build the systems [41,42]. The histidine 233 was exchanged for leucine (H233L), histidine 398 was converted into a proline (H398P), and the arginine 553 was replaced with a proline (R553P). All systems, wild-type (WT), and the mutants H233L, H398P, and R553P were solvated using the TIP3P water model. The solvated systems were ionized and neutralized with NaCl at a concentration of 150 mM. The water box size was 120 × 119 × 199 Å. Ionized systems were minimized, equilibrated, and run for 1 µs of simulation. MD simulations were performed with AMBER (San Francisco, CA, USA) using the ff19SB force field for proteins and GAFF for PIP_2_ ligand [43,44,45]. The pressure was fixed at 1 atm, and the temperature was kept constant at 310.15 K through the Langevin thermostat with the isobaric-isothermal (NPT) ensemble. Each condition was simulated in three replicates and ran under periodic boundary conditions. After completing the simulations, the subsequent objective was to ascertain the molecular factors associated with the inactivity of PLCζ enzyme mutants. To achieve this, various geometric and energetic stability parameters were computed.

### 4.2. PIP_2_ Mean Square Displacement and Diffusion Coefficient

The Mean Square Displacement (MSD) is a measure of the average squared distance that the molecules travel over time within the binding site. It quantifies the molecule’s movement and diffusion, providing insights into its dynamic behavior and interactions with the enzyme during the trajectory. On the other hand, the Diffusion Coefficient (D) of a molecule in the binding site of an enzyme, derived from the MSD, quantifies the rate at which the molecule diffuses within the binding site. It is calculated from the slope of the MSD versus time. MSD and D were computed from the Diffusion Coefficient Tool [46] of VMD v.1.9.3 software (Urbana-Champaign, IL, USA) [47]. This analysis was performed on a PIP_2_ ligand placed into the binding site of the PLCζ enzyme. Each calculation involves the quantification of the three axes (x, y, z). The τ value was assigned as the default parameter considering the whole trajectory.

### 4.3. Binding Free Energy

The protein-ligand affinity energy was computed throughout the entire trajectory at regular intervals of every 10 ns, utilizing the end-point Molecular Mechanics-Generalized Born Surface Area (MM-GBSA) method of AMBER22 (San Francisco, CA, USA) [48]. This approach accurately estimates the binding free energy between protein and ligand in a molecular system by combining molecular mechanics calculations to represent the protein-ligand complex with a continuum solvent model, the Generalized Born model, to account for solvation effects. By considering both the energetic contributions from the molecular mechanics force field and the solvent interactions, MM-GBSA aims to provide insights into the thermodynamics of binding.

### 4.4. Protein-Ligand Contacts

Contact types and frequencies were calculated using the GetContacts application (https://getcontacts.github.io/ (accessed on 13 March 2025)). We computed the intermolecular ligand-side chain hydrogen bonds using a donor-acceptor distance < 3.5 Å and an angle of 180°–70°. Likewise, we calculated the ligand-protein salt bridge using a distance cutoff of < 4.0 Å. The van der Waals (vdw) was assessed by employing this equation:

|AB| < Rvdw(A) + Rvdw(B) + 0.5

where A and B are any non-hydrogen atoms.

### 4.5. Root Mean Square Fluctuations

The Root Mean Square Fluctuations (RMSF) is a measure of the average deviation of each residue from its average position over time. It quantifies the flexibility and mobility of different parts of the enzyme, indicating which regions are more dynamic and which are more rigid. The enzyme’s RMSF profile was calculated using an in-house Tcl script and run in VMD v.1.9.3 software (Urbana-Champaign, IL, USA).

### 4.6. Ca^2+^ Ion Mean Square Displacement and Diffusion Coefficient

To comprehend the mechanisms involving the Ca^2+^ cofactor and potential chelating effects exhibited by certain mutants, we quantified the ion’s MSD and D throughout the simulation, mirroring the methodology employed for the PIP_2_ ligand.

### 4.7. Radial Distribution Function

The Radial Distribution Function (RDF) of water molecules surrounding an ion throughout a simulation measures how the density of water molecules varies as a function of distance from the ion. It provides insights into the spatial organization and structure of the water molecules around the ion. RDF was calculated by averaging the distribution of water molecules at various distances from the ion using the plugin “Radial Pair Distribution Function g(r)” of VMD v.1.9.3 software (Urbana-Champaign, IL, USA).

### 4.8. Root Mean Square Deviation

The Root Mean Square Deviation (RMSD) of an enzyme through an MD trajectory measures the average deviation of the enzyme’s residue positions from a reference structure over time. It quantifies the enzyme’s overall conformational changes and structural stability during the simulation. RMSD was calculated along the trajectory using the “RMSD trajectory” tool within VMD v.1.9.3 (Urbana-Champaign, IL, USA). Each domain’s backbone of the PLCζ protein was individually delineated as follows: EF-hands (residues 35 to 145), XY (residues 155 to 465), and C2 (residues 466 to 589).

### 4.9. Intramolecular Hydrogen Bonds per Domain

The number of intra-domain hydrogen bonds was determined using the VMD’s “Hydrogen Bonds” tool. The tool employs default donor-acceptor distance and angle parameters of 3.0 Å and 20°, respectively. The hydrogen bond numbers were quantified in each domain independently.

### 4.10. Ion Coordination

To evaluate the protein residues and numbers of water molecules coordinating the Ca^2+^ ion, we built an in-house Tcl script that quantifies the protein residues and water molecules within a coordination sphere of 3.5 Å of radius along the simulation time.

### 4.11. Radius of Gyration

The radius of gyration (Rg) measures the enzyme’s compactness and overall shape, providing comprehension of the enzyme’s folding, unfolding, and conformational stability during the simulation. The Rg was evaluated with an in-house Tcl script, separately assessing the three PLCζ domains (EF-hands, XY, and C2).

### 4.12. Statistics

Statistical analysis was performed using GraphPad Prism v.10.4.0 (Boston, MA, USA). The Kolmogorov–Smirnov test was used to determine the data distribution. ANOVA and the Kruskal–Wallis test were used to compare parametric and non-parametric data, respectively. The data were plotted using RStudio software v.2024.09.0+375.

## 5. Conclusions

Our molecular dynamics simulations provide valuable insights into the structural consequences of PLCζ mutations associated with male infertility. Our findings suggest that mutations such as H233L and H398P disrupt PIP_2_ and Ca^2+^ binding and identify residues within the PLCζ structure that stabilize their binding. Understanding the molecular basis of these mutations can aid in developing targeted therapeutic strategies to overcome male infertility. Importantly, these function-abrogating mutations could be the basis for developing non-hormonal contraceptive methods.

## Data Availability

The original contributions presented in this study are included in the article/Supplementary Materials. Further inquiries can be directed to the corresponding author(s).

## Supplementary Materials

The following supporting information can be downloaded at: https://www.mdpi.com/article/10.3390/ijms26104706/s1.

## Abbreviations

The following abbreviations are used in this manuscript:

ART	Assisted reproductive technology
DAG	Diacylglycerol
GAFF	The General Amber force field
IP_3_	Inositol 1,4,5-triphosphate
IP_3_R	Inositol 1,4,5-triphosphate receptor
MD	Molecular dynamics simulations
MM-GBSA	Molecular Mechanics-Generalized Born Surface Area
MSD	Mean square deviation
NPT	Isobaric-isothermal ensemble
PDB	Protein Data Bank
PIP_2_	Phosphatidylinositol 4,5-biphosphate
PLCζ	Phospholipase Cζ
PLC∂	Phospholipase C∂
PM	Plasma membrane
RDF	Radial distribution function
Rg	Radius of gyration
RMSD	Root mean square deviation
RMSF	Root mean square fluctuations
vdw	Van der Waals
VMD	Visual molecular dynamics
WT	Wild-type
