# Supplementary material for: Structural Implications of H233L and H398P Mutations in Phospholipase Cζ: A Full-Atom Molecular Dynamics Study on Infertility-Associated Dysfunctions
Source: Int J Mol Sci. 2025 May 14;26(10):4706. doi: 10.3390/ijms26104706 (PMC12111915; doi:10.3390/ijms26104706)
Supplement: Supplementary file 1 [file ijms-26-04706-s001.zip › ijms-3557613-supplementary.pdf]

Supplementary Material

Structural implications of H233L and H398P mutations in phospholipase Cζ: A full-atom molecular dynamics study on infertility-associated dysfunctions

Fernando Hinostroza, Sofía Albornoz-Muñoz, SebastiánVergara, Gabriela Urra, Ingrid Araya-Duran, Rafael Fissore, Fernando Danilo González-Nilo, Daniel Bustos, Ingrid Carvacho.

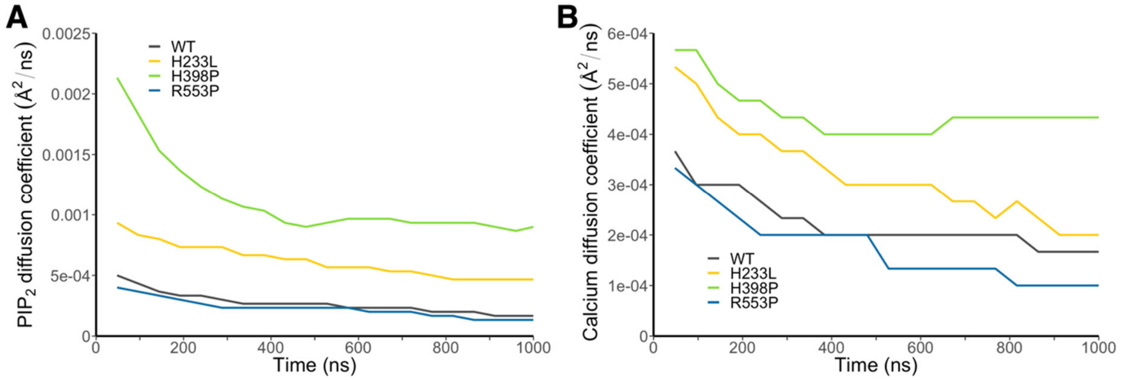

Figure S1: *H233L and H398P mutations increase PIP2 and Ca^{2+} diffusion coefficient*. Coefficient diffusion of **(A)** PIP2 and **(B)** Ca^{2+} for all PLCζ mutants H233L (yellow), H398P (green) and R553P (blue) and WT (black).

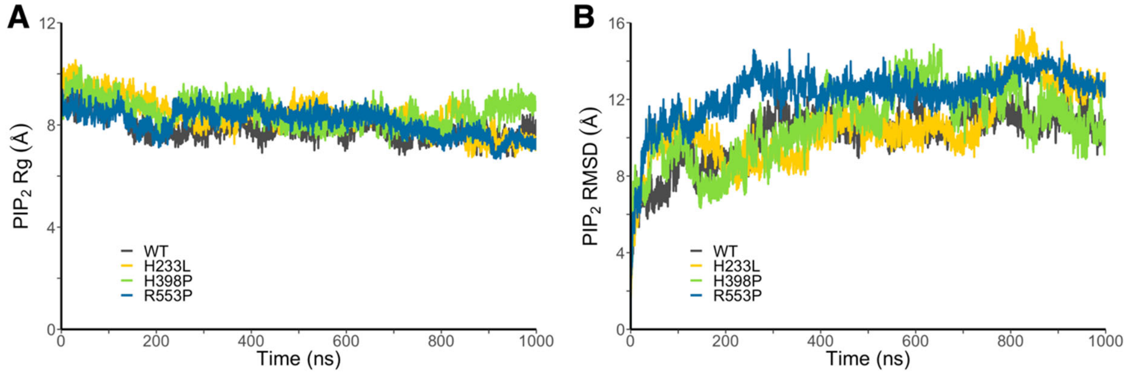

Figure S2: *PLCζ mutations do not change PIP_2 configuration*. The position of PIP_2 within the PLCζ structure of the WT (black) and mutants H233L (yellow), H398P (green) and R553P (blue) was evaluated. **A**. Radius of gyration (Rg) and **(B)** root mean square deviation (RMSD) of PIP_2.
